# Respiratory viruses in individuals with a high frequency of animal exposure in southern and highland Vietnam

**DOI:** 10.1002/jmv.25640

**Published:** 2019-12-12

**Authors:** Tu Thi Kha Nguyen, Tue Tri Ngo, Phuc My Tran, Tam Thi Thanh Pham, Hang Thi Ty Vu, Ny Thi Han Nguyen, Guy Thwaites, Anna‐Maija K. Virtala, Olli Vapalahti, Stephen Baker, Tan Le Van

**Affiliations:** ^1^ Faculty of Medicine University of Helsinki Helsinki Finland; ^2^ Oxford University Clinical Research Unit Ho Chi Minh City Vietnam; ^3^ Dong Thap Provincial Center for Disease Control Dong Thap Province Vietnam; ^4^ Centre for Tropical Medicine and Global Health Oxford University Oxford United Kingdom; ^5^ Department of Veterinary Biosciences, Faculty of Veterinary Medicine University of Helsinki Helsinki Finland; ^6^ Department of Virology and Immunology HUSLAB, Helsinki University Hospital Helsinki Finland; ^7^ Department of Medicine University of Cambridge Cambridge United Kingdom

**Keywords:** asymptomatic, cohort study, viral etiology, respiratory disease, Vietnam, zoonoses

## Abstract

Active surveillance for zoonotic respiratory viruses is essential to inform the development of appropriate interventions and outbreak responses. Here we target individuals with a high frequency of animal exposure in Vietnam. Three‐year community‐based surveillance was conducted in Vietnam during 2013‐2016. We enrolled a total of 581 individuals (animal‐raising farmers, slaughterers, animal‐health workers, and rat traders), and utilized reverse transcription‐polymerase chain reaction to detect 15 common respiratory viruses in pooled nasal‐throat swabs collected at baseline or acute respiratory disease episodes. A respiratory virus was detected in 7.9% (58 of 732) of baseline samples, and 17.7% (136 of 770) of disease episode samples (*P* < .001), with enteroviruses (EVs), rhinoviruses and influenza A virus being the predominant viruses detected. There were temporal and spatial fluctuations in the frequencies of the detected viruses over the study period, for example, EVs and influenza A viruses were more often detected during rainy seasons. We reported the detection of common respiratory viruses in individuals with a high frequency of animal exposure in Vietnam, an emerging infectious disease hotspot. The results show the value of baseline/control sampling in delineating the causative relationships and have revealed important insights into the ecological aspects of EVs, rhinoviruses and influenza A and their contributions to the burden posed by respiratory infections in Vietnam.

## Highlights

- We reported the detection of common respiratory viruses in individuals with a high frequency of animal exposure in Vietnam, an emerging infectious disease hotspot. The results show the value of baseline/control sampling in delineating the causative relationships and have revealed important insights into the ecological aspects of EVs, rhinoviruses and influenza A and their contributions to the burden posed by respiratory infections in Vietnam.

## 1 INTRODUCTION

Annually, acute respiratory tract infections are responsible for more than 3 million deaths worldwide.^1^ In Vietnam, a developing country in Southeast Asia, mortality attributed to acute respiratory infections accounted for half of that attributed to the other infectious diseases combined in 2016.^1^

Viruses are regarded as the most common causes of acute respiratory diseases, and some emerging respiratory diseases as the Middle East respiratory syndrome (MERS) and severe acute respiratory syndrome (SARS), both related to coronaviruses (CoVs), are listed in the WHO's List of Blueprint priority diseases^2^ because of their pandemic potential. While the reported patterns of the etiological agents vary between geographic locations and age groups, generally, respiratory syncytial virus (RSV)‐A, RSVB, influenza A virus, influenza B virus, adenovirus (ADV), enterovirus (EVs); human metapneumovirus (MPV), human rhinovirus (HRV), parainfluenza virus (PIV)1‐4, human CoV (including subtypes OC43 and NL63), human bocavirus (BoV) and parechovirus (PEV) are the most common viruses detected in respiratory samples worldwide.^3^, ^4^, ^5^, ^6^, ^7^, ^8^, ^9^ Of these, influenza A virus, influenza B virus, and CoV have been reported as the most common viruses detected in people over 5 years old,^10^, ^11^, ^12^, ^13^ while RSV and PIVs have been regarded as the leading causes of respiratory infections in children under 5 years old in South East Asia.^3^, ^14^, ^15^

Zoonotic infections are of global concern, and approximately 60% of known infectious diseases in humans are of zoonotic origin.^16^ In addition, Southeast Asia, including Vietnam, is one of the hotspots of emerging infectious diseases. Indeed, many of the recent respiratory outbreaks are linked with zoonotic viruses as SARS‐CoV,^17^ avian influenza A virus H5N1,^18^ pandemic influenza A virus subtype H1N1,^19^ and more recently MERS‐CoV,^20^ with the majority being first reported in Asia. Collectively, active surveillance for (novel) zoonotic viruses in this vulnerable part of the world is of both medical and public health significance. As such, for the detection of novel zoonotic viruses in humans and animals, during 2012‐2015 the Vietnam Initiative on Zoonotic InfectiONS (VIZIONS) project, consisting of the various hospital‐ and community‐based studies, was conducted across Vietnam.^21^, ^22^ Herein, we focus our analysis on a community‐based study, which was designed to capture the cross‐species transmission events of zoonotic viruses among individuals with a high risk of zoonotic infections in southern and highland Vietnam. In this study, our aim was to describe the frequency of common respiratory viruses in clinical samples collected from these individuals, later called cohort members, at baseline and when a respiratory disease episode was reported during the study period.

## 2 METHODS

### 2.1 Study design and inclusion criteria

This study was a part of the High‐Risk Sentinel Cohort (HRSC) study which was a community‐based component of the VIZIONS project.^21^ The HRSC study was first commenced in June 2013 in Dong Thap and then in February 2014 in Dak Lak. These are provinces located in southern and highland of Vietnam, respectively, representing two different geographic areas in Vietnam.

Animal‐raising farmers, animal health workers, and slaughterers were eligible to be enrolled in the study since these are common occupations in rural Vietnam with frequent occupational exposure to animals. Rat traders in Dong Thap were additionally recruited due to the commonality of this occupation in this locality. The animal‐raising farmers accounted for about two‐third of the population with occupational exposure to animals in these study provinces.

On the basis of the animal farm census, letters were sent out to invite potential participants to attend an introductory meeting. The consent forms were then obtained from those who were willing to join the HRSC study. For each farmer household, up to four members having the highest frequency of working with animals were recruited. The slaughterers were recruited from all local central abattoirs or slaughter points. The animal‐health workers and rat traders were selected by convenience. Consequently, a total of 581 individuals (median age in year, 38; range, 2‐89), including 415 (71.4%) animal‐raising farmers, 100 (11.7%) slaughterers, 61 (10.5%) animal‐health workers, and 5 (1.8%) rat‐traders, were recruited. Each cohort members were followed up annually for up 3 years since recruitment.

### 2.2 Data collection

Annually, to establish the baseline data (ie, no disease episode reported), the cohort members were interviewed, and clinical specimens, including rectal, pooled nasal, and throat swabs and blood were also collected from each interviewee. These baseline data were collected from all cohort members, except for the farmers, for which only one person mostly working with animals per household was interviewed and sampled.

During the study period, whenever getting illness (diarrhea and respiratory infection) defined as any signs/symptoms of respiratory tract infections (eg, sneezing, coughing or sore throat), plus fever (≥38°C), the cohort members informed the local study teams. Within 48 hours, the site study doctors made a visit to the participant houses and collected information about animal exposures, associated symptoms, and medication. In addition, clinical specimens, including blood, and (when relevant) rectal‐ or pooled nasal and throat swabs were collected. All the specimens were stored at −80°C until analysis. Here, we focused on respiratory episodes. As such, only pooled nasal‐throat swabs of each individual were analyzed.

### 2.3 Respiratory virus detections by real‐time polymerase chain reaction analysis

To detect common respiratory viruses in pooled nasal and throat swabs, we first isolated total nucleic acid (NA) from patient samples using MagNA Pure 96 platform (Roche Diagnostics, Mannheim, Germany), following the manufacturer's instructions. The NA output was then eluted in 50 µL of elution buffer and immediately screened for respiratory viruses using multiplex real‐time polymerase chain reaction (RT‐PCR) assays.

The RT‐PCR assays used in the present study were derived from previous publications,^23^, ^24^, ^25^, ^26^ which captured 15 common respiratory viruses and a wide range of their subtypes, including RSVA, RSVB; influenza A virus, influenza B virus, ADV; EVs; MPV; HRV; PIV‐1, PIV‐2, PIV‐3, PIV4; CoV subtype OC43 and NL63; BoV and PEV.^23^, ^24^, ^25^ Influenza A virus‐positive samples were further tested for (zoonotic) subtypes, including H3, H1N1pdm09, H1, and avian/H5 25, 26 (primer and probe sequences are listed in Table S2). All the RT‐PCR reactions were carried in a LightCycler 480 Instrument II (96‐wells) (Roche Molecular Systems, Inc).

### 2.4 Data analysis

The data were analyzed by STATA software, version 12.0.^27^ The pairwise comparisons of categorical variables were calculated by Pearson's *χ* ^2^ test (or Fisher exact test when the sample size was less than five in any of the cells of a contingency table) or two‐sample *t* test with equal variances. The errors of multiple comparisons were corrected by the Bonferroni method.^28^ *P* ≤ .05 was considered the significance. EpiTools^29^ were used to calculate 95% confidence intervals for the odds ratio. The rat traders (n = 5) were excluded from these tests because of an insufficient sample size.

### 2.5 Ethics

The HRSC study was approved by the Oxford Tropical Research Ethics Committee (OxTREC) in the United Kingdom, and by the Ethics Committees of Dong Thap Hospital, Dak Lak Hospital, the sub‐Departments of Animal Health in Dong Thap and Dak Lak, and the Hospital of Tropical Diseases in Ho Chi Minh City in Vietnam. Written informed consent was obtained from each study participant.

## 3 RESULTS

### 3.1 Collection of respiratory swabs and reports of disease episodes

The detailed characteristics of the cohort members are briefly summarized in Table 1. Approximately half (51.1%; 297 of 581) of the study population was annually interviewed during 2013‐2015, corresponding to a total of 829 interviews conducted (291, 273, and 265 interviews in 2013, 2014, and 2015, respectively) (Table 1). Consequently, 732 pooled nasal‐throat swabs were collected at these annual interviews for respiratory virus detection. Herein, these samples were considered as baseline samples.

**Table 1:** Baseline characteristics

	All	Dak Lak	Dong Thap	*P* value^a^
Occupation	N = 581	N = 299	N = 282	.012
Farmers, n (%)	415 (71)	201 (67)	214 (76)	**.021**
Animal‐health workers, n (%)	61 (10)	31 (10)	30 (11)	.915
Slaughterers, n (%)	100 (17)	67 (22)	33 (12)	**.001**
Rat traders, n (%)	5 (1)	0	5 (2)	
Median age (range), y	38 (2‐89)	39 (2‐89)	38 (4‐76)	.995^b^
Age groups				
≤15, n (%)	59 (10)	24 (8)	35 (12)	.080
≥16, n (%)	522 (90)	275 (92)	247 (88)	
Sex ratio (male/female)	1.2 (322/259)	1.1 (157/142)	1.4 (165/117)	.146
No. of cohort members interviewed annually for baseline^c^	N = 297	N = 162	N = 135	
1st year, n (%)	291 (98)	162 (100)	129 (96)	**.042**
2nd year, n (%)	273 (92)	150 (93)	123 (91)	.114
3rd year, n (%)	265 (89)	147 (91)	118 (87)	.077
No. of cohort members reporting respiratory illness	N = 386	N = 219	N = 167	
1st year, n (%)	227 (59)	154 (70)	73 (44)	**<.001**
2nd year, n (%)	193 (50)	109 (50)	84 (50)	.088
3rd year, n (%)	151 (39)	67 (31)	84 (50)	**.043**
No. of reported respiratory episodes^d^	N = 812	N = 394	N = 418	
1st year, n (%)	317 (39)	183 (46)	134 (32)	**.017**
2nd year, n (%)	317 (39)	129 (33)	188 (45)	**.001**
3rd year, n (%)	178 (22)	82 (21)	96 (23)	.758

^a^ *P* value (Pearson's *χ* ^2^ or Fisher exact test) of the difference between Dak Lak and Dong Thap.

^b^ *t Test*.

^c^ At these follow‐up time points, a respiratory sample was collected from each individual.

^d^ A total of 770 samples were collected and included in polymerase chain reaction analysis, with 314, 281, and 175 samples in first, second, and third years, respectively.

Over the 3‐year period, 66.4% (386 of 581) of the cohort members reported having respiratory infections, corresponding to a total of 812 respiratory episodes (Table 1), or an average of 2.1 episodes per reporting individual, and 1.4 (812/581) episodes per individual among all cohort members. The slaughterers (225/100) were more likely to have respiratory diseases than the animal‐health workers (92/61) and the farmers (491/415) (*P* < .003). In total, of the 812 reported respiratory episodes, 770 pooled nasal‐throat swabs were collected for respiratory virus detection.

### 3.2 Frequency of respiratory viruses detected at baseline and the disease episodes

Evidence of a respiratory virus by RT‐PCR analysis was documented in 7.9% (58 of 732) of samples collected at the baseline, and 17.7% (136 of 770) of samples collected when a respiratory disease episode was reported (*P* < .001) (Table 2). In addition, mixed infections were recorded in 2 (0.3%) and 7 (0.9%) samples collected at baseline and disease episodes, respectively (Table 2).

**Table 2:** Number (percentage) of cohort members with detected viruses from tested pooled nasal and throat swabs

	Whole study				1st year				2nd year				3rd year			
	Baseline (N = 732)	Disease episode (N = 770)	*P* value	OR (95% CI)	Baseline (N = 290)	Disease episode (N = 314)	*P* value	OR (95% CI)	Baseline (N = 240)	Disease episode (N = 281)	*P* value	OR (95% CI)	Baseline (N = 202)	Disease episode (N = 175)	*P* value	OR (95% CI)
EVs	29 (4.0)	67 (8.7)	<.001	2.3 (1.5‐3.6)	2 (0.7)	12 (3.8)	.013	5.7 (1.3‐26)	10 (4.2)	40 (14.2)	<.001	3.8 (1.8‐7.8)	17 (8.4)	15 (8.6)	.96	1 (0.5‐2.1)
HRV	5 (0.7)	32 (4.2)	<.001	6.3 (2.4‐16)	2 (0.7)	21 (6.7)	<.001	10.3 (2.4‐44)	1 (0.4)	9 (3.2)	.024	7.9 (1‐63)	2 (1.0)	2 (1.1)	1	1.2 (0.2‐8.3)
Influenza A virus	14 (1.9)	18 (2.3)	.568	1.2 (0.6‐2.5)	4 (1.4)	4 (1.3)	1	1 (0.2‐3.7)	6 (2.5)	11 (3.9)	.365	1.6 (0.6‐4.4)	4 (2.0)	3 (1.7)	1	0.8 (0.2‐3.9)
H3	3 (21.4)	12 (66.7)	.016	7.3 (1.5‐36)	0	1 (0.3)	1	NA	0	10 (3.6)	.002	NA	3 (1.5)	1 (0.6)	.63	0.4 (0‐3.7)
H1‐seasonal	0	0	NA	NA	0	0	NA	NA	0	0	NA	NA	0	0	NA	NA
H1‐pan09	0	0	NA	NA	0	0	NA	NA	0	0	NA	NA	0	0	NA	NA
H5	0	0	NA	NA	0	0	NA	NA	0	0	NA	NA	0	0	NA	NA
ADV	1 (0.1)	9 (1.2)	.021	8.6 (1.1‐68)	1 (0.3)	4 (1.3)	.375	3.7 (0.4‐33)	0	2 (0.7)	.502	4.3 (0.2‐90)	0	3 (1.7)	.1	8.2 (0.4‐160)
CoV^a^	8 (1.1)	7 (0.9)	.72	0.8 (0.3‐2.3)	1 (0.3)	4 (1.3)	.375	3.7 (0.4‐33)	6 (2.5)	3 (1.1)	.314	3.2 (1.3‐8)	1 (0.5)	0	1	NA
RSVA	0	3 (0.4)	.25	NA	0	3 (1.0)	.250	NA	0	0	NA	NA	0	0	NA	NA
MPV	0	2 (0.3)	.5	NA	0	1 (0.3)	1	NA	0	1 (0.4)	1	NA	0	0	NA	NA
RSVB	1 (0.1)	2 (0.3)	1	1.9 (0.2‐21)	1 (0.3)	0	1	0.3 (0‐7.5)	0	2 (0.7)	.502	NA	0	0	NA	NA
Influenza B virus	0	2 (0.3)	0.5	NA	0	0	NA	NA	0	0	NA	NA	0	2 (1.1)	.22	NA
PIV4	0	1 (0.1)	1	NA	0	0	NA	NA	0	1 (0.4)	1	NA	0	0	NA	NA
BoV	2 (0.3)	1 (0.1)	.615	0.5 (0‐5.3)	1 (0.3)	0	.48	NA	0	1 (0.4)	1	NA	1 (0.5)	0	1	NA
(+) ≥1 virus	58 (7.9)	136 (17.7)	<.001	2.5 (1.8‐3.5)	12 (4.1)	45 (14.3)	<.001	3.9 (2‐7.5)	22 (9.2)	67 (23.8)	<.001	3.1 (1.8‐5.2)	24 (12)	24 (13.7)	.64	1.2 (0.6‐2.1)
(+) ≥2 viruses^b^	2 (0.3)	7 (0.9)	.18	3.3 (0.7‐16)	0	2 (0.6)	.5	4.6 (0.2‐97)	1 (0.4)	4 (1.4)	.38	3.4 (0.4‐31)	1 (0.5)	1 (0.6)	1	1.2 (0‐18.6)

*Note:* The OR was the disease episodes vs baseline. *P* values were calculated by Pearson's *χ* ^2^ or Fisher exact test.

Abbreviations: ADV, adenovirus; BoV, bocavirus; CI, confidence interval; CoV, coronavirus; EV, enterovirus; HRV, human rhinovirus; OR, odds ratio; PEV, parechovirus; PIV, parainfluenza virus; RSV, respiratory syncytial virus.

^a^ Subtype OC43 and NL63.

^b^ Including one EVs‐BoV and one influenza A virus‐CoV in the baseline, and one AdV‐influenza B virus, one BoV‐influenza A virus, two EVs‐influenza A virus, one EVs‐RSVB, one EVs‐HRV and one EVs‐AdV‐CoV in the disease episodes PIV‐1, ‐2, ‐3, and PEV were not detected in all samples.

Of the detected viruses, EVs, HRV and influenza A virus were the most common viruses detected in samples collected at both baseline and disease episodes, followed by ADV and CoV (Table 2). There were significant differences in the frequencies of EVs, HRV and ADV detected in the two groups; 29 of 732 (4%) at baseline vs 67 of 770 (8.7%) at disease episodes (*P* < .001) for EVs, 5 of 732 (0.7%) vs 32 of 770 (4.2% (*P* < .001) for HRV, and 1 of 732 (0.1%) vs 9 of 770 (1.2%; (*P* = .021) for ADV (Table 2). In addition, of the influenza A virus RT‐PCR positive cases, subtype H3 was detected at a higher frequency at disease episodes than at baseline, 66.7% (12 of 15) vs 21.4% (3 of 14), *P* = .016. Remaining influenza A virus‐positive cases were RT‐PCR negative for specific RT‐PCR for the other tested subtypes (H1N1pdm09, H1N1, and H5) (Table 2).

### 3.3 Clinical signs/symptoms of cohort members in acute respiratory diseases with the detected viruses

For the altogether 770 reported respiratory episodes, cough and sneezing were the most common symptoms recorded, present in 76% (585 of 770) and 74.7% (575 of 770) of cases, respectively, followed by sore throat (65.3%; 503 of 770), headache (51.4%; 396 of 770), body aches (41.8%; 322 of 770), and dyspnea (7.4%; 57 of 770) (Table 3). In addition, gastrointestinal symptoms were recorded in 7.3% (56 of 770), but watery diarrhea was more often recorded in cohort members without a virus detected than in those with a positive finding, 52 of 634 (8.2%) vs 4 of 136 (2.9%), *P* = .029 (Table 3).

**Table 3:** Number (and percentage) for demographics and clinical characteristics of cohort members reporting respiratory episodes

	Tested samples (N = 770)	Positive samples (N = 136)	Negative samples (N = 634)	*P* value	OR (95% CI)	EVs (N = 67)	HRV (N = 32)	Influenza A virus (N = 18)	ADV (N = 9)	CoV^a^ (N = 7)
Age ≤ 15	60 (7.8)	15 (11)	45 (7.1)	.121	1.6 (0.9‐3)	8 (11.9)	3 (9.4)	2 (11.1)	2 (22.2)	2 (28.6)
Fever	770 (100)	136 (100)	634 (100)	NA	NA	67 (100)	32 (100)	18 (100)	9 (100)	7 (100)
Cough	585 (76.0)	102 (75.0)	483 (76.2)	.770	0.9 (0.6‐1.4)	49 (73)	23 (72)	15 (83)	8 (89)	6 (86)
Sneezing	575 (74.7)	105 (77.2)	470 (74.1)	.455	1.2 (0.8‐1.8)	50 (75)	25 (78)	15 (83)	6 (67)	7 (100)
Sore throat	503 (65.3)	90 (66.2)	413 (65.1)	.818	1.1 (0.7‐1.6)	46 (69)	16 (50)	15 (83)	4 (44)	5 (71)
Dyspnea	57 (7.4)	8 (5.9)	49 (7.7)	.456	0.8 (0.3‐1.6)	4 (6)	2 (6)	0	0	0
Headache	396 (51.4)	70 (51.5)	326 (51.4)	.991	1 (0.7‐1.5)	30 (45)	20 (63)	11 (61)	3 (33)	3 (43)
Body aches	322 (41.8)	48 (35.2)	274 (43.2)	.089	0.7 (0.5‐1.1)	21 (31)	11 (34)	9 (33)	0	2 (29)
Watery diarrhea	56 (7.3)	4 (2.9)	52 (8.2)	.029	0.3 (0.1‐1)	2 (3)	2 (6)	0	0	0
Nausea	55 (7.1)	8 (5.9)	47 (7.4)	.529	0.8 (0.4‐1.7)	3 (4)	1 (3)	2 (11)	1 (11)	1 (14)
Antibiotic use^b^, ^c^	199 (25.8)	37 (27.2)	162 (25.6)	.689	1.1 (0.7‐1.7)	18 (26.9)	5 (15.6)	4 (22.2)	1 (11.1)	2 (28.6)

*Note:* Four patients have no data on gender and age. *P* values were calculated by Pearson's *χ* ^2^ or Fisher exact test. The difference in each viral infection inducing each clinical symptom was not significant (*P* > .05). The OR was the disease “positive samples” vs “negative samples”.

Abbreviations: ADV, adenovirus; CI, confidence interval; CoV, coronavirus; EV, enterovirus; HRV, human rhinovirus; OR, odds ratio.

^a^ Subtype OC43 and NL63.

^b^ The antibiotic use of the patients from the first symptoms to the incidence interview/sampling.

^c^ Antibiotic types used Cephalosporin, Amoxicillin, Clarithromycin, Ampicillin, Augmentin, Azithromycin, Chloramphenicol, Ciprofloxacin, Erythromycin, Ofloxacin, Spiramycin.

Of the virus‐positive cases, watery diarrhea was only recorded in those positive for EVs and HRV, whilst sore throat was predominantly recorded in those positive for influenza A virus. Otherwise, there were considerable similarities in age and clinical presentations of cohort‐member groups who were positive for different viruses (Table 3).

### 3.4 The frequency of respiratory viruses detected by provinces

To assess the differences in the frequencies of respiratory viruses under investigation between Dong Thap and Dak Lak, which represent the two distinct geographic localities in Vietnam, we stratified the data for these two individual provinces (Table 4). Subsequently, EVs, HRV and influenza A virus remained the leading viruses detected in the tested samples from these provinces, while the detection rates of EVs and HRV in disease episode samples collected in Dong Thap were significantly higher than that in Dak Lak (11.1% [42 of 379] vs 6.4% [25 of 391]; *P* = .021, and 6.1% [23 of 379] vs 2.3% [9 of 391], *P* = .009, respectively). In Dong Thap, EVs and HRV were significantly more often detected in samples collected at disease episode than at baseline; *P* < .001 for both EVs and HRV. In Dak Lak, no significant differences were found (Table 4).

**Table 4:** Number (percentage) of cohort members with different detected viruses at baseline and disease episodes in Dak Lak and Dong Thap

	Dak Lak				Dong Thap				Baseline (Dak Lak vs Dong Thap)		Disease episodes (Dak Lak vs Dong Thap)	
	Baseline (N = 434)	Disease episode (N = 391)	*P* value	OR (95% CI)^a^	Baseline (N = 298)	Disease episode (N = 379)	*P* value	OR (95% CI)^a^	*P* value	OR (95% CI)^b^	*P* value	OR (95% CI)^b^
EVs	18 (4.1)	25 (6.4)	.147	1.6 (0.9‐2.9)	11 (3.7)	42 (11.1)	<.001	3.3 (1.6‐6.4)	0.756	1.1 (0.5‐2.4)	.021	0.6 (0.3‐0.9)
HRV	4 (0.9)	9 (2.3)	.112	2.5 (0.8‐8.3)	1 (0.3)	23 (6.1)	<.001	19 (2.6‐143)	0.653	2.8 (0.3‐25)	.009	0.4 (0.2‐0.8)
Influenza A virus	6 (1.4)	9 (2.3)	.324	1.7 (0.6‐4.8)	8 (2.7)	9 (2.4)	.798	0.9 (0.3‐2.3)	0.206	0.5 (0.2‐1.5)	.947	1 (0.4‐2.5)
H3	0	4 (44.4)	.103	NA	3 (37.5)	8 (88.9)	.050	13.3 (1.1‐166)	0.067	0.1 (0‐1.9)	.256	0.5 (0.1‐1.6)
H1‐seasonal	0	0	NA	NA	0	0	NA	NA	NA	NA	NA	NA
H1‐pan09	0	0	NA	NA	0	0	NA	NA	NA	NA	NA	NA
H5	0	0	NA	NA	0	0	NA	NA	NA	NA	NA	NA
ADV	1 (0.2)	5 (1.3)	.107	5.6 (0.7‐48)	0	4 (1.1)	.135	NA	1	2.1 (0.1‐50)	1	1.2 (0.3‐4.6)
CoV^c^	4 (0.9)	3 (0.8)	1	0.8 (0.2‐3.7)	4 (1.3)	4 (1.1)	.736	0.8 (0.2‐3.2)	0.722	0.7 (0.2‐2.8)	.721	0.7 (0.2‐3.3)
RSVA	0	0	NA	NA	0	3 (0.8)	.26	NA	NA	NA	.119	0.1 (0‐2.7)
MPV	0	0	NA	NA	0	2 (0.5)	.506	NA	NA	NA	.242	0.2 (0‐4)
RSVB	1 (0.2)	0	1	NA	0	2 (0.5)	.506	NA	1	2.1 (0.1‐50)	.242	0.2 (0‐4)
Influenza B virus	0	0	NA	NA	0	2 (0.5)	.506	NA	NA	NA	.242	0.2 (0‐4)
PIV4	0	0	NA	NA	0	1 (0.3)	1	NA	NA	NA	.492	0.3 (0‐8)
BoV	1 (0.2)	0	1	NA	1 (0.3)	1 (0.3)	1	0.8 (0.1‐12)	1	0.7 (0‐11)	.492	0.3 (0‐8)
(+) ≥1 virus	33 (7.6)	48 (12.3)	.024	1.7 (1.1‐2.7)	25 (8.4)	88 (23.2)	<.001	3.3 (2.1‐5.3)	0.699	0.9 (0.5‐1.5)	<.001	0.5 (0.3‐0.7)
(+) ≥2 viruses^d^	2 (0.5)	2 (0.5)	.917	1.1 (0.2‐7.9)	0	5 (1.3)	.071	NA	0.517	3.4 (0.2‐72)	.28	0.4 (0.1‐2)

*Note:* Other viral pathogens were not showed as they were detected in less than 10 samples. *P* values were calculated by Pearson's *χ* ^2^ or Fisher exact test

Abbreviations: ADV, adenovirus; CI, confidence interval; CoV, coronavirus; EV, enterovirus; HRV, human rhinovirus; OR, odds ratio.

^a^ The OR was the disease episodes vs. baseline.

^b^ The OR was Dak Lak and Dong Thap.

^c^ Subtype OC43 and NL63.

^d^ Including one EVs‐BoV and one influenza A virus‐CoV in the baseline, and one AdV‐influenza B virus, one BoV‐influenza A virus, two EVs‐influenza A virus, one EVs‐RSVB, one EVs‐HRV, and one EVs‐AdV‐CoV in the disease episodes.

### 3.5 Temporal and seasonal differences in the frequency of detection of respiratory viruses

There were some fluctuations in the detection of the most common viruses (especially EVs and HRV; Table 2) over the study period. Of particular note was the significant increase in the frequency of EVs from baseline to disease episodes in the first 2 years (from 0.7% at baseline to 3.8% at disease episodes in the first year, and from 4.2% to 14.2% in the second year, respectively) (Table 2). In year 3, the detection of EVs remained high but was comparable in samples collected at baseline (8.4%, 17 of 202) and disease episodes (8.6%, 15 of 175) (Table 2). In contrast to EVs, there was a downward trend of HRV detection over time, while the frequency of influenza A virus was relatively stable over the 3‐year period (Table 2).

**Figure 1:**
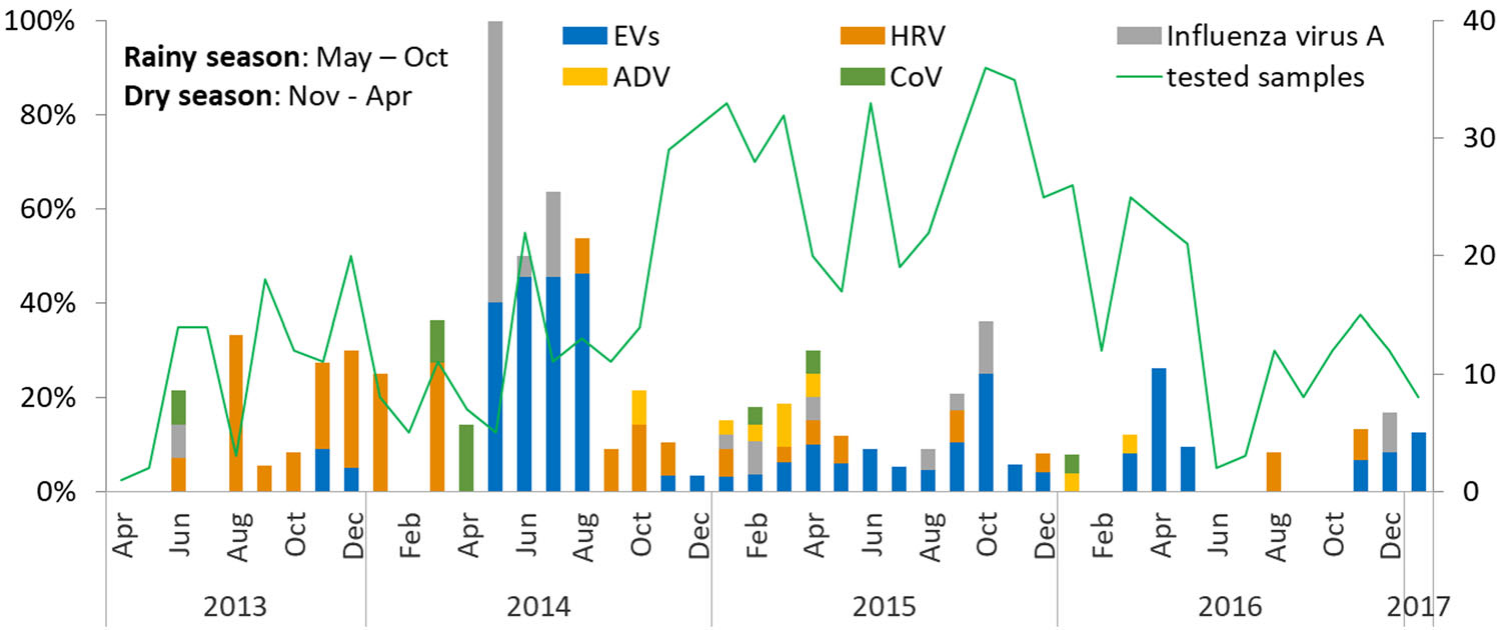
The seasonal distribution of symptomatic EVs‐, HRV‐, influenza A virus‐, ADV‐, and CoV (subtype OC43 and NL63)‐infected cases detected by RT‐PCR assay. The bars show the proportion of the viruses detected among total samples tested (the line chart) each month. EVs and influenza A virus were more likely detected in the rainy season than in the dry season (*P* = .002 and *P* = .023, respectively), while the ADV detections were more frequent in the dry season as compared with the rainy one (*P* = .044). There was no significant difference in the detections of HRV and CoV (subtype OC43 and NL63) between dry and rainy seasons (*P* = .333 and .227, respectively). ADV, adenovirus; CoV, coronavirus; EV, enterovirus; HRV, human rhinovirus; RT‐PCR, real‐time polymerase chain reaction

In terms of seasonality, overall, there were some clear trends in the seasonality of the most common viruses (especially EVs and influenza A virus, Figure 1). More specifically, EVs and influenza A virus were significantly more often found in rainy season (May‐October) than in dry season (November‐April); the detection rates were 12.2% (43 of 353) vs 5.8% (24 of 417) (*P* = .002) for EVs, and 3.7% (13 of 353) vs 1.2% (5 of 417), *P* = .023 for influenza A virus, respectively. In contrast to EVs and influenza A virus, ADV was more often found in the dry season than in the rainy season (1.9%, 8 of 417 vs 0.3%, 1 of 353, *P* = .044) (Figure 1).

### 3.6 Animal exposure

Overall, the cohort members were exposed to a wide range of animals, including 11 types of exotic animals and 11 types of domestic animals, within ≤1 month prior to the disease episode (n = 770) (Table S1). There was no difference in the patterns of animal exposure among cohort members who were positive for the predominant viruses (EVs, HRV, and influenza A virus). The numbers of the remaining viruses were insufficient to informatively assess their associations with age, seasonality, clinical presentation, and animal exposure.

## 4 DISCUSSION

Here we describe the frequency of common human viruses causing acute respiratory infections in people with high exposure to animals in Dong Thap (Southern) and Dak Lak (Highland) provinces. We showed that EVs, HRV and influenza A virus were the predominant viruses detected in respiratory samples of the cohort members in both localities and that their detection rates were significantly higher in respiratory samples collected at respiratory disease episodes than in those collected at baseline. In addition, the results have also revealed important insights into the ecological characteristics of these predominant viruses. More specifically, our analysis shows that EVs and influenza A virus were more often found in the rainy season (from May to October), and there were fluctuations in the detection of EVs and HRV over time, while influenza A virus activity was relatively stable over the study period, suggesting that these viruses may have interacted with the immune landscape of the study population.

Although viral detection in upper respiratory samples like pooled nasal and throat swabs may merely reflect the carriage of such viruses in these body cavities, a higher detection frequency in samples collected at disease episodes than at baseline suggests an association between the detected viruses and the reported respiratory episodes. As such, the high frequency of EVs and HRV detected in samples collected at disease episodes in the present study further expand our knowledge about the clinical burden posed by viruses of the genus *Enterovirus* in Vietnam. Indeed, an outbreak of enterovirus associated diseases like hand foot and mouth disease (HFMD) have been frequently reported in Vietnam and Asia since 1997.^30^, ^31^ Likewise, enteroviruses have been reported to be one of the leading causes of central nervous system infections and respiratory illness in Vietnam.^5^, ^32^, ^33^ In addition, in line with the observed cyclical epidemic patterns of HFMD in Vietnam and Asia,^30^, ^31^ for which the underlying mechanism remains unknown, the fluctuations in the detection EVs and HRV over the study period and between Dong Thap and Dak Lak suggest an interplay between the pathogens and the proportion of susceptible individuals in respective provinces.

The higher detection of influenza A virus subtype H3 in samples collected at the disease episodes than in those collected at baseline points to the association between subtype H3 with respiratory illness in Vietnam. In contrast to the prevalence of influenza A virus subtype H3, the result showing an overall comparable prevalence of influenza A virus in both sample groups suggests that there is a high level of asymptomatic infection of influenza A virus in the general population, in agreement with previous reports.^34^, ^35^ The difference in sensitivities between RT‐PCR assays used may explain our failure to identify the specific influenza A virus subtypes in the remaining pan‐influenza A virus RT‐PCR positive samples.

The low prevalence or absence of respiratory viruses like PIVs, PEV, RSVA, and RSVB in the present study may be attributed to the age structure of the present cohort. Indeed, while, these viral species are well‐established agents of (respiratory) infections in children, and to some extent in elderly people (eg, in case of PIVs),^3^, ^10^, ^14^, ^15^, ^36^, ^37^, ^38^ over 92% of the respiratory disease episodes reported in this study were among cohort members aging ≥16 years. In terms of seasonal distribution of the predominant viruses as EVs, influenza A virus, HRV and ADV, our report supports previous findings.^39^, ^40^, ^41^, ^42^, ^43^

Our overall RT‐PCR yield of 17.7% of viral agents in respiratory samples of the cohort members with the majority age from 16 years or above is in agreement with the diagnostic yields of previous studies.^44^, ^45^, ^46^, ^47^, ^48^, ^49^ The results suggest that it is probably because adults have acquired substantial immunity during their life, leading to the rapid clearance of the infecting viruses from their respiratory tract, thereby shortening the duration of viral shedding.

Our study has some limitations. First, no human subjects without animal exposure were recruited as controls. Therefore, we were unable to assess the effect (if any) of animal exposure on the frequency of the respiratory disease incidence, as well as the observed viral patterns. Second, despite a holistic effort, nonviral agents as bacterial pathogens were not tested. Third, a slight decrease in sensitivity of the multiplex RT‐PCR platforms used in the present study as compared with that of the corresponding monoplex RT‐PCRs have previously been reported,^23^ which may in part explain the absence of respiratory viruses in some of the tested samples. Collectively, future studies should explore if unbiased pan‐pathogen assays, namely metagenomic next‐generation sequencing‐based approach could improve the etiological detection in patients presenting with respiratory infection; the usefulness of this approach has already been shown for other diseases worldwide, especially in low‐ and middle‐income countries like Vietnam.^50^

## 5 CONCLUSION

We reported the detection of common respiratory viruses in individuals with a high frequency of animal exposure in two distinct geographic regions in Vietnam, representing one of the broad‐range, prospective and controlled screenings for viral etiologies of respiratory illnesses in people with unique animal contacts in a setting where zoonotic emerging infections are likely to occur. The results show the value of baseline/control sampling in analyzing causative relationships and have revealed important insights into the ecological aspects of EVs, HRV and influenza A and their contributions to the burden posed by respiratory infections in Vietnam.

## THE VIZIONS CONSORTIUM MEMBERS

The VIZIONS Consortium members (alphabetical order by surname) from the Oxford University Clinical Research Unit are Bach Tuan Kiet, Stephen Baker, Alessandra Berto, Maciej F. Boni, Juliet E. Bryant, Bui Duc Phu, James I. Campbell, Juan Carrique‐Mas, Dang Manh Hung, Dang Thao Huong, Dang Tram Oanh, Jeremy N. Day, Dinh Van Tan, H. Rogier van Doorn, Duong An Han, Jeremy J. Farrar, Hau Thi Thu Trang, Ho Dang Trung Nghia, Hoang Bao Long, Hoang Van Duong, Huynh Thi Kim Thu, Lam Chi Cuong, Le Manh Hung, Le Thanh Phuong, Le Thi Phuc, Le Thi Phuong, Le Xuan Luat, Luu Thi Thu Ha, Ly Van Chuong, Mai Thi Phuoc Loan, Behzad Nadjm, Ngo Thanh Bao, Ngo Thi Hoa, Ngo Tri Tue, Nguyen Canh Tu, Nguyen Dac Thuan, Nguyen Dong, Nguyen Khac Chuyen, Nguyen Ngoc An, Nguyen Ngoc Vinh, Nguyen Quoc Hung, Nguyen Thanh Dung, Nguyen Thanh Minh, Nguyen Thi Binh, Nguyen Thi Hong Tham, Nguyen Thi Hong Tien, Nguyen Thi Kim Chuc, Nguyen Thi Le Ngoc, Nguyen Thi Lien Ha, Nguyen Thi Nam Lien, Nguyen Thi Ngoc Diep, Nguyen Thi Nhung, Nguyen Thi Song Chau, Nguyen Thi Yen Chi, Nguyen Thieu Trinh, Nguyen Thu Van, Nguyen Van Cuong, Nguyen Van Hung, Nguyen Van Kinh, Nguyen Van Minh Hoang, Nguyen Van My, Nguyen Van Thang, Nguyen Van Thanh, Nguyen Van Vinh Chau, Nguyen Van Xang, Pham Ha My, Pham Hong Anh, Pham Thi Minh Khoa, Pham Thi Thanh Tam, Pham Van Lao, Pham Van Minh, Phan Van Be Bay, Maia A. Rabaa, Motiur Rahman, Corinne Thompson, Guy Thwaites, Ta Thi Dieu Ngan, Tran Do Hoang Nhu, Tran Hoang Minh Chau, Tran Khanh Toan, Tran My Phuc, Tran Thi Kim Hong, Tran Thi Ngoc Dung, Tran Thi Thanh Thanh, Tran Thi Thuy Minh, Tran Thua Nguyen, Tran Tinh Hien, Trinh Quang Tri, Vo Be Hien, Vo Nhut Tai, Vo Quoc Cuong, Voong Vinh Phat, Vu Thi Lan Huong, Vu Thi Ty Hang, and Heiman Wertheim; from the Center for Immunity, Infection, and Evolution, University Of Edinburgh: Carlijn Bogaardt, Margo Chase‐Topping, Al Ivens, Lu Lu, Dung Nyugen, Andrew Rambaut, Peter Simmonds, and Mark Woolhouse; from The Wellcome Trust Sanger Institute, Hinxton, United Kingdom: Matthew Cotten, Bas B. Oude Munnink, Paul Kellam, and My Vu Tra Phan; from the Laboratory of Experimental Virology, Department of Medical Microbiology, Center for Infection and Immunity Amsterdam (CINIMA), Academic Medical Center of the University of Amsterdam, Amsterdam, the Netherlands: Martin Deijs, Lia van der Hoek, Maarten F. Jebbink, and Seyed Mohammad Jazaeri Farsani; and from Metabiota, CA: Karen Saylors and Nathan Wolfe.

## CONFLICT OF INTERESTS

The authors declare that there are no conflict of interests.

## Supporting information

Supporting information
