# Supplementary material for: Respiratory viruses in individuals with a high frequency of animal exposure in southern and highland Vietnam
Source: J Med Virol. 2019 Dec 12;92(8):971–81. doi: 10.1002/jmv.25640 (PMC7228379; doi:10.1002/jmv.25640)
Supplement: Supplementary file 1 — Supporting information [file JMV-92-971-s001.doc]

**Supplementary materials**

**Supplementary Table 1**: Number of animals (percentages) that the cohort members exposed to less than one month prior to the onset of the disease episodes.

|  | **All the patients, N=770,  exposed via:** | | | **The patients with EVs infections, N=67, exposed via:** | | | **The patients with HRV infections, N=32, exposed via:** | | | **The patients with influenza A virus infections, N=18, exposed via:** | | |
| --- | --- | --- | --- | --- | --- | --- | --- | --- | --- | --- | --- | --- |
|  | **Presence^&^** | **Handling^#^** | **Cooking^** | **Presence** | **Handling** | **Cooking** | **Presence** | **Handling** | **Cooking** | **Presence** | **Handling** | **Cooking** |
| Chicken (n (%)) | 475 (61.7) | 224 (29.1) | 97 (12.6) | 37 (55.2) | 9 (13.4) | 5 (7.5) | 18 (56.3) | 4 (12.5) | 3 (9.4) | 12 (66.7) | 6 (33.3) | 4 (22.2) |
| Dog | 430 (55.8) | 146 (19) | 0 | 34 (50.7) | 11 (16.4) |  | 19 (59.4) | 3 (9.4) |  | 10 (55.6) | 5 (27.8) |  |
| Pig | 277 (36) | 74 (9.6) | 3 (0.4) | 25 (37.3) | 7 (10.4) |  | 15 (46.9) | 3 (9.4) |  | 8 (44.4) | 4 (22.2) |  |
| Duck | 230 (29.9) | 71 (9.2) | 1 (0.1) | 15 (22.4) | 5 (7.5) |  | 11 (34.4) | 2 (6.3) |  | 9 (50) | 4 (22.2) |  |
| Cat | 160 (20.8) | 64 (8.3) | 19 (2.5) | 15 (22.4) | 4 (6) | 1 (1.5) | 7 (21.9) | 2 (6.3) |  | 5 (27.8) | 1 (5.6) | 1 (5.6) |
| Wild pig* | 133 (17.3) | 77 (10) | 4 (0.5) | 9 (13.4) | 3 (4.5) |  | 4 (12.5) | 2 (6.3) |  | 4 (22.2) | 1 (5.6) |  |
| Deer* | 99 (12.9) | 52 (6.8) | 6 (0.8) | 4 (6) | 3 (4.5) |  | 8 (25) | 1 (3.1) |  | 2 (11.1) | 2 (11.1) |  |
| Cattle | 66 (8.6) | 37 (4.8) | 4 (0.5) | 1 (1.5) | 1 (1.5) |  | 2 (6.3) |  |  | 1 (5.6) |  |  |
| Other wild bird* | 62 (8.1) | 35 (4.5) | 0 | 6 (9) | 4 (6) |  |  |  |  | 1 (5.6) | 1 (5.6) |  |
| Muscovy duck | 60 (7.8) | 32 (4.2) | 0 | 6 (9) | 3 (4.5) |  | 2 (6.3) |  |  |  |  |  |
| Goose | 60 (7.8) | 26 (3.4) | 2 (0.3) | 4 (6) | 2 (3) |  | 3 (9.4) | 1 (3.1) |  | 1 (5.6) |  |  |
| Porcupine* | 49 (6.4) | 18 (2.3) | 4 0.5) | 4 (6) |  |  |  |  |  | 1 (5.6) | 1 (5.6) |  |
| Goat | 45 (5.8) | 34 (4.4) | 1 (0.1) | 2 (3) | 1 (1.5) |  | 1 (3.1) | 1 (3.1) |  | 3 (16.7) | 1 (5.6) |  |
| Pigeon | 34 (4.4) | 21 (2.7) | 0 | 2 (3) | 1 (1.5) |  | 2 (6.3) | 1 (3.1) |  |  |  |  |
| Rabbit | 17 (2.2) | 16 (2.1) | 1 (0.1) |  | 1 (1.5) |  |  |  |  | 1 (5.6) |  |  |
| Pangolin* | 13 (1.7) | 0 | 0 | 2 (3) |  |  |  |  |  |  |  |  |
| Bamboo rat* | 5 (0.6) | 2 (0.3) | 0 |  |  |  |  |  |  |  |  |  |
| Rat* | 3 (0.4) | 3 (0.4) | 0 |  |  |  | 1 (3.1) | 1 (3.1) |  |  |  |  |
| Squirrel* | 2 (0.3) | 1 (0.1) | 0 |  |  |  |  |  |  |  |  |  |
| Civet* | 1 (0.1) | 0 | 0 |  |  |  |  |  |  |  |  |  |
| Bat* | 1 (0.1) | 0 | 0 |  |  |  |  |  |  |  |  |  |
| Bear* | 1 (0.1) | 1 (0.1) | 0 |  |  |  |  |  |  | 1 (5.6) | 1 (5.6) |  |

*^*^ indicate the exotic animals*

***^&^****the exposure to the animals at the household backyard or the vicinity where they were living*

*^#^the exposure by animal slaughtering or handling*

*^ the exposure by animal cooking and preparation.*

**Supplementary Table 2**: Primer and probe sequences of the (RT-)PCRs used

| **Primer-probe name** | **Sequence** |
| --- | --- |
| mp1 FluB F | TCGCTGTTTGGAGACACAAT |
| mp1 FluB R | TTCTTTCCCACCGAACCA |
| mp1 FluB probe | CYAN500-AGAAGATGGAGAAGGCAAAGCAGAACT-BHQ1 |
| mp1 ENT F | GGCCCTGAATGCGGCTAAT |
| mp1 ENT R | GGGATTGTCACCATAAGCAGCC |
| mp1 ENT probe | 6-FAM-CGGAACCGACTACTTTG+G+GT-BBQ |
| mp1 ADV F | CAGGACGCCTCGGRGTAYCTSAG |
| mp1 ADV R | GGAGCCACVGTGGGRTT |
| mp1 ADV probe | LCRED670-CGGGTCTGGTGCAGTTTGCCCGC-BBQ |
| mp2 MPV F | AGCTTCAGTCAATTCAACAGAAG |
| mp2 MPV R | CCTGCAGATGTYGGCATGT |
| mp2 MPV probe | FAM-TGTTGTGCGGCAGTTTTCAGACAATGC-BBQ1 |
| mp2 Rhi F | AGSCTGCGTGGCKGCC |
| mp2 Rhi R | ACACGGACACCCAAAGTAGT |
| mp2 Rhi probe | CYAN500-TCCTCCGGCCCCTGAATGYGGCTAAYC-BHQ1 |
| mp2 EAV F | CATCTCTTGCTTTGCTCCTTAG |
| mp2 EAV R | AGCCGCACCTTCACATTG |
| mp2 EAV probe | Cy5-CGCTGTCAGAACAACATTATTGCCCAC-BHQ3 |
| mp3 PIV1 F | ATCTCATTATTACCYGGACCAAGTCTACT |
| mp3 PIV1 R | CATCCTTGAGTGATTAAGTTTGATGAATA |
| mp3 PIV1 probe | CYAN500-AGGATGTGTTAGAYTACCTTCATTATCAATTGGTGATG-BHQ1 |
| mp3 PIV2 F | CTGCAGCTATGAGTAATC |
| mp3 PIV2 R | TGATCGAGCATCTGGAAT |
| mp3 PIV2 probe | LCRED610-AGCCATGCATTCACCAGAAGCCAGC-BBQ |
| mp3 PIV3 F | ACTCTATCYACTCTCAGACC |
| mp3 PIV3 R | TGGGATCTCTGAGGATAC |
| mp3 PIV3 probe | FAM-AAgggACCACgCgCTCCTTTCATC-BHQ1 |
| mp3 PIV4 F | GATCCACAGCAAAGATTCAC |
| mp3 PIV4 R | GCCTGTAAGGAAAGCAGAGA |
| mp3 PIV4 probe | Cy5-TATCATCATCTgCCAAATCggCAA-BHQ3 |
| mp4 Cor F1 | GGTGGYTGGGAYGATATGTTACG |
| mp4 Cor R1 | KRTTTGGCATAGCACGATCACA |
| mp4 Cor 1 probe | 6-FAM-ATGTTGACAAYCCTGTWCTTATGGGTTGGG-BBQ |
| mp4 Cor F2 | GCTRAGCATGATTTCTTTACTTGG |
| mp4 Cor R2 | CARTYTTKTTCATCAAAGTTACGCA |
| mp4 Cor 2 probe | 6-FAM-CAGARTCATTTATGGTAATGTTAGTAGACA-BBQ |
| mp4 PEV F | CTGGGGCCAAAAGCCA |
| mp4 PEV R | GGTACCTTCTGGGCATCCTTC |
| mp4 PEV probe | Cy5-AAACACTAgT+T+gT+AW+gg+C+CC-BHQ3 |
| mp4 Boc F | CAAATCTCTTCTGGCTACACG |
| mp4 Boc R | CTCTGCGATCTCTATATTGAAGG |
| mp4 Boc probe | Cyan500-ATgTTgCCgCCAgTAACTCCACC-BHQ1 |
| RSV-MTH_F | GGATTCTACCATATATTGA |
| RSV-MTH_R | GAAGTKAGGAAATTGAGT |
| RSVprobe A-LNA | Cyan500-5'-ca+Aaagc+At+Cat+Ta+Tt+Atc+Ttt -BHQ1 |
| RSVprobe B-LNA | FAM- 5’- caaaagcatcattg+Ct+Gtc+Att  - BHQ1 |
| CDC FluA-F (Universal) | GAC CRA TCC TGT CAC CTC TGA C |
| CDC FluA-R(Universal) | AGG GCA TTY TGG ACA AAK CGT CTA |
| CDC A/H3-F | AAG CAT TCC YAA TGA CAA ACC |
| CDC A/H3-R | ATT GCR CCR AAT ATG CCT CTA GT |
| SwH1-09 F | GTTACCCAGGAGATTTCATCGA |
| SwH1-09 R | CATGCTGCCGTTACACCTTTG |
| CDC A/H5a-F | TGG AAA GTR TAA RAA ACG GAA CGT |
| CDC A/H5a-R | YGC TAG GGA RCT CGC CAC TG |
| CDC A/H5b-F | GGA ATG CCC CAA ATA TGT GAA ATC AA |
| CDC A/H5b-R | CTC CCC TGC TCA TTG CTA TGG T |
| CDC FluA probe (Universal) | FAM-TGC AGT CCT CGC TCA CTG GGC ACG-BHQ1 |
| CDC A/H3 probe | FAM-CAG GAT CAC ATA TGG GSC CTG TCC CAG-BHQ1 |
| Inhouse-SwH1-09 probe | FAM-AAGTTCATGGCCCAATCATGACTCGA-BHQ1 |
| CDC A/H5a probe 1 | TGA CTA CCC GCA G”T”A TTC AGA AGA AGC AAG ACT AA |
| CDC A/H5a probe 2 | CAA CTA TCC GCA G”T”A TTC AGA AGA AGC AAG ATT AA |
| CDC A/H5b probe | TAC CCA TAC CAA CCA “T”CT ACC ATT CCC TGC CAT |
